# Transcranial Magnetic Stimulation for Post-traumatic Stress Disorder

**DOI:** 10.3389/fpsyt.2022.701348

**Published:** 2022-05-31

**Authors:** Amber N. Edinoff, Tanner L. Hegefeld, Murray Petersen, James C. Patterson, Christopher Yossi, Jacob Slizewski, Ashley Osumi, Elyse M. Cornett, Adam Kaye, Jessica S. Kaye, Vijayakumar Javalkar, Omar Viswanath, Ivan Urits, Alan D. Kaye

**Affiliations:** ^1^Department of Psychiatry and Behavioral Medicine, Louisiana State University Health Science Center Shreveport, Shreveport, LA, United States; ^2^Creighton University School of Medicine, Omaha, NE, United States; ^3^Department of Anesthesiology, Louisiana State University Shreveport, Shreveport, LA, United States; ^4^Department of Pharmacy Practice, Thomas J. Long School of Pharmacy and Health Sciences, University of the Pacific, Stockton, CA, United States; ^5^Department of Neurology, Louisiana State University Shreveport, Shreveport, LA, United States; ^6^College of Medicine-Phoenix, University of Arizona, Phoenix, AZ, United States; ^7^Department of Anesthesiology, Creighton University School of Medicine, Omaha, NE, United States; ^8^Valley Anesthesiology and Pain Consultants–Envision Physician Services, Phoenix, AZ, United States; ^9^Southcoast Health, Southcoast Physicians Group Pain Medicine, Wareham, MA, United States

**Keywords:** PTSD, Transcranial Magnetic Stimulation, anxiety, neurobiological treatment, Post-traumatic stress disorder

## Abstract

Post-traumatic stress disorder (PTSD) is a psychiatric disorder that causes significant functional impairment and is related to altered stress response and reinforced learned fear behavior. PTSD has been found to impact three functional networks in the brain: default mode, executive control, and salience. The executive control network includes the dorsolateral prefrontal cortex (DLPFC) and lateral PPC. The salience network involves the anterior cingulate cortex, anterior insula, and amygdala. This latter network has been found to have increased functional connectivity in PTSD. Transcranial Magnetic Stimulation (TMS) is a technique used in treating PTSD and involves stimulating specific portions of the brain through electromagnetic induction. Currently, high-frequency TMS applied to the left dorsolateral prefrontal cortex (DLPFC) is approved for use in treating major depressive disorder (MDD) in patients who have failed at least one medication trial. In current studies, high-frequency stimulation has been shown to be more effective in PTSD rating scales posttreatment than low-frequency stimulation. The most common side effect is headache and scalp pain treated by mild analgesics. Seizures are a rare side effect and are usually due to predisposing factors. Studies have been done to assess the overall efficacy of TMS. However, results have been conflicting, and sample sizes were small. More research should be done with larger sample sizes to test the efficacy of TMS in the treatment of PTSD. Overall, TMS is a relatively safe treatment. Currently, the only FDA- approved to treat refractory depression, but with the potential to treat many other conditions.

## Introduction

Post-traumatic stress disorder (PTSD) is classified as a “trauma and stressor-related disorder” in the Diagnostic and Statistical Manual (DSM-5) (1). About 7–9% of the general population develop PTSD in their lifetime (2). Patients diagnosed with PTSD often have poorer social support and higher rates of occupational, financial, and health problems (3). In addition, these patients are at increased risk for attempting suicide and have more frequent marital difficulties and intimate relationship problems (4–6). This manuscript aims to look at PTSD as well as Transcranial Magnetic Stimulation (TMS) which is an alternative treatment for PTSD symptoms.

### Diagnostic Definition and Clinical Presentation

The following criteria are required for the diagnosis of PTSD (1). To meet criterion A, a person must have been exposed to one or more traumatic events. If criterion A is met, the person must present with clustered symptoms that satisfy criteria B-E. The symptom clusters are intrusion, avoidance, negative alterations in cognitions and mood, and alterations in arousal and reactivity. An intrusive symptom can be distressing memories, recurrent distressing dreams, dissociative reactions (e.g., flashbacks), or intense or prolonged psychological distress when exposed to reminders of the trauma. Next, a person must present with avoidance symptoms related to the traumatic event. This includes avoidance of internal reminders (thoughts and memories) or active avoidance of external reminders (people, places, activities, etc.). A person must present with two symptoms which could include negative alterations in cognitions and mood. This includes an inability to remember an important aspect of the traumatic event; persistent and exaggerated negative thoughts about oneself, others or the world, persistent and distorted cognitions about the cause or consequences of the event; persistent negative emotions; diminished interest in activities, feeling detached from others, and/or a persistent inability to experience positive emotions. Finally, a person must present with two arousal symptoms which can include irritable behavior and angry outbursts, reckless or self-destructive behavior, hypervigilance, exaggerated startle response, problems with concentration, and/or sleep disturbances. These symptoms must be present for 1 month or longer following the trauma.

The presentation of PTSD is variable between individuals but must include one example from each domain. Symptoms present within 3 months and persist for longer than 1 month and may be intermittent. Other than these 4 categories of symptoms, individuals may also experience somatic manifestations that resemble physical illness and can affect various systems such as musculoskeletal, nervous, cardiac, respiratory, and gastrointestinal (7). Following a trauma, it is normal to experience distress, but individuals with PTSD are not able to decrease their fear of certain triggers and confront certain stimuli leading to the development of avoidance strategies in order to prevent distressing emotions and stimuli. These feelings and behavioral changes are found in each of the four clusters of symptoms.

### Epidemiology

PTSD has a lifetime prevalence of 7–12% in the US (8). As one of the only psychiatric conditions with an identifiable cause, PTSD is often seen in those who have experienced trauma. These include combat, personal assault, natural disasters, motor vehicle accidents, rape, childhood physical and sexual abuse, loss of loved ones, and medical crises (9). Compared to civilians, military personnel have a higher incidence of overall PTSD prevalence, with up to 30% of veterans experiencing PTSD and 15% continuing to experience symptoms 10 years after the conclusion of war (10). These numbers tend to vary between studies. However, the consensus is that there is a larger percentage of those who have combat and military associated traumatic experience with PTSD compared to the civilian population.

Most people do not develop PTSD after exposure to trauma. While risk factors for PTSD do play a role, the most significant factor is the severity of the trauma. Some events such as rape or direct combat are associated with rates of PTSD up to 50% (21). Other differences within the population are the prevalence in women compared to men. Despite fewer exposures to trauma, women are at higher risk with a lifetime prevalence of 20.4% compared to men at 8.2% (9).

### Risk Factors

#### Genetic Factors

Twin studies have shown genetic influences play a role in the development of PTSD, accounting for a 30-72% of the vulnerability to develop PTSD (22–24). In addition, twin studies have demonstrated the comorbidity of PTSD with other mental disorders such as Major Depressive Disorder, Generalized Anxiety Disorder, Panic Disorder, and Substance Use Disorder (25). Candidate gene studies have largely focused on the genes involved in the Hypothalamic-Pituitary-Adrenal axis, with over 50 gene variants linked with PTSD (26, 27). However, candidate gene studies have shown mixed results and attention has turned toward Genome Wide Association Studies, which have identified common variants and novel loci, not previously identified by biologic studies (27, 28). Research has started to look at epigenetic processes as well, mostly focused on DNA methylation, which has identified a number of implicated genes (29, 30).

#### Risk Factors Associated With PTSD Beyond Neural Networks

Not everyone who experiences trauma develops PTSD; however, those who do are vulnerable and possess risk factors. Directly associated with the ability to handle the stress response is the idea of resilience, which is the ability to adapt successfully in response to adversity, trauma, or significant threat. The lack of resilience and presence of various risk factors are involved in the development of PTSD. The risk factors are broken into three categories including pre-trauma, peri-trauma and post-trauma (7).

Pre-traumatic risk factors include characteristics such as demographics, health history, neurobiological, and cognitive characteristics. Females are twice as likely to be affected and have symptoms for a longer duration and poorer quality of life. Age also plays a role with the lowest incidence found in those older than 60 and the highest incidence found in individuals aged 45–59. IQ has been found to be inversely correlated with the risk of PTSD and high IQ is potentially a protective factor in trauma-exposed individuals. Sexual minorities are at greater risk related to higher rates of early childhood abuse and greater exposure to violence and to traumatic events. Other pre-trauma risk factors include psychopathology and familial psychiatric history, specifically the risk is increased in anxious individuals and early onset mood disorders (7). Lastly, certain races and ethnicities have been found to have a higher association with PTSD with Blacks and Hispanics with the highest lifetime prevalence and Asians with the lowest (31).

Peritraumatic risk factors are critical in assessing the risk of developing PTSD and include trauma type and severity. There is a higher association of PTSD with sexual assault or physical injury. Associated with this is how one perceives the trauma. If an individual deems that the incident involved is a true threat to one's life or led to significant losses, they are at greater risk of developing PTSD. For example, the military previously utilized debriefing sessions immediately after trauma in an attempt to promote emotional processing. Although these sessions were created in an attempt to prevent maladaptive emotional outcomes, by encouraging sudden recollection of the event there was an observed increase in PTSD as these debriefing sessions led to re-traumatization (32). Additionally, included in the peri-traumatic period is how one processes their trauma. An individual is less likely to develop PTSD if they perceive their experience as having ended vs. if they feel a continued threat and are unable to disengage from their traumatic experience (7).

Lastly, there are posttraumatic factors that predispose individuals to developing PTSD. Psychosocial factors are associated with coping with stress and include optimism, cognitive flexibility, and active coping skills. Cognitive flexibility or rigidity is important in the processing of traumatic events and can alter the likelihood of resilient outcomes vs. psychopathology. This processing pertains to how one interprets trauma and is able to reevaluate their perception and experience of the trauma. Following a trauma, if one generalizes their experience this may lead to inflexibility and development of PTSD symptoms. Access to needed resources such as psychological first aid, protection from further harm, connection to required services all are protective against the development of PTSD. Addressing an individual's needs and concerns through compassion and support is protective as well as allowing for the normalization of one's emotional response (33). The presence of social support networks is important; the lack of a social support system in addition to ongoing life stress can increase the risk of developing PTSD. Physical activity has been found to boost resilience and is considered to be a protective factor. The presence of mature defense mechanisms such as altruism produces psychological and physiological benefits assists in the post-traumatic period and can confer resilience (7, 34).

Pathophysiology: Neural Networks, Relevant Brain Structures Further Explained, HPA Axis and Sympathetic Nervous System

#### Pathophysiology: Neural Networks

PTSD is believed to be related to abnormal stress response and fear learning, that in turn impact three cerebrocortical networks: the default mode, the executive control, and salience networks. PTSD involves the derangement of a multitude of pathways and structures that normally work in synchrony to properly process stress. Each neural network involves different brain structures, has diverse responsibilities, and is either disrupted or enhanced in PTSD. The default mode network is comprised of regions including the MPFC, PCC, PPC, and TC. The default mode network is the network of brain regions that is active when the brain is at “rest” (not sleeping), and involves interoceptive processing of information that pertains to oneself as well as episodic memory. This network has been found to have reduced connectivity in PTSD, observed in relation to memory dysfunction and processing of fear (2). The executive control network includes the DLPFC and lateral PPC, which are also disrupted in PTSD (11). Normally, this network regulates executive function, particularly emotion regulation and working memory. Lastly, the salience network involves the anterior cingulate cortex, anterior insula, and amygdala. It has been found to have increased functional connectivity in PTSD. Since this network is responsible for the detection of environmental stimuli, this upregulation in activity is observed as increased threat detection (35). The network relationships that vary between individuals may play a role in the severity of symptoms and individual experience (35). In addition, these varying networks are useful as a clinical measure for the evaluation of PTSD symptoms and can predict response to treatment (36).

A recent neuroimaging study found that there were aberrant connections in a treatment-resistant form of PTSD. They found that a subgroup in their study displayed both aberrant connections in the ventral attention network (VAN) as seen on fMRI and impaired verbal memory on a word list learning task (37). This phenotype could be used to predict a poor response to psychotherapy. These authors also used focal TMS and electroencephalography they identified alterations in the neurosignal flow in the VAN that was evoked by direct stimulation of that network (37). They concluded that their findings identified neurobiological mechanisms in a subgroup of patients with PTSD that could be responsible for the poor response to psychotherapy.

#### Pathophysiology: Relevant Brain Structures Further Explained

Another approach to the pathophysiology of PTSD involves understanding the different brain regions involved in the stress response. Generally, the current understanding of PTSD is hyperactivity of deeper structures and hypoactivity of the prefrontal cortex. These deeper structures include the amygdala and hippocampus and are involved in memory consolidation and fear circuitry (2, 11, 38). The prefrontal cortex can be subdivided into regions and the ventromedial prefrontal cortex (VMPFC) and DLPFC have been implicated in PTSD. The VMPFC plays a role in emotional regulation and suppression of the fear response and the DLPFC in executive functioning (2, 11).

In PTSD, the amygdala is hyperactive in response to negative stimuli and neutral stimuli, while exhibiting a lesser response to positive stimuli. The amygdala is an important contributor to the reward system and decision making in terms of behavior (39). There are connections that run from the amygdala to the nucleus accumbens, which is the main reward system responsible for reinforcing behaviors (39). This finding explains the amygdala's role in PTSD, specifically how emotional processing and reward reinforcement create abnormal behavioral and emotional changes in response to triggers; these abnormal behaviors and emotional changes are learned and then reinforced overtime. Additionally, the amygdala has been found to be coactivated with the hippocampus when exposed to negative stimuli. The hippocampus was also found to be enhanced in response to negative content and this increased activity was found to be related to worse memory performance. Altogether, this finding may not only explain the overgeneralization of fear in PTSD, as there is enhanced hippocampus activity, but also altered memory accuracy.

Lastly, certain regions of the prefrontal cortex are important players in the pathophysiology of PTSD. The medial structures of the cortex are activated in response to emotional triggers. The VMPFC is heavily connected to the amygdala and acts to regulate emotion. The activity of the VMPFC is diminished in PTSD leading to increased activation and reactivity of the amygdala since it a role in the inhibitory control over the amygdala (40). Additionally, the VMPFC can alter the original fear that led to the development of PTSD and instead override it with safe memories (9). The DLPFC is involved in complex cognitive and behavioral functions. These functions include working memory, attention control, decision-making, and behavioral organization. There is an indirect connection involved in regulation of the mood network comprised of the amygdala and hippocampus. In PTSD, the DLPFC is hypoactive, and similar to the VMPFC leads to hyper reactivity of the amygdala (41).

#### Pathophysiology: HPA Axis and Sympathetic Nervous System

The underlying symptoms that result from the deranged stress response found in PTSD are connected to a complex integrated adaptive system of signaling molecules comprised of hormones, neurotransmitters, and neuropeptides. The stress response is comprised of the HPA axis and sympathetic nervous system. The HPA axis is regulated by negative feedback, however, in PTSD this feedback loop is altered due to the presence of low basal cortisol levels and raised catecholamine levels (38). There is ongoing research attempting to explain the cause of the HPA axis derangements including the existence of genetic variants of glucocorticoid receptors (36). Other studies have investigated the causes of hypocortisolism in relation to adrenal response and activity (31). The HPA axis is modified due to the presence of chronic stress and should continue to be a research subject for both explaining the pathophysiology of PTSD and its utility as a potential treatment target.

### Current Treatment of PTSD

Current treatment for PTSD includes a wide variety of psychotherapies and pharmacotherapy that is applied based on clinical presentation, compliance, and severity. According to the Veteran's Affairs/Department of Defense (VA/DoD), American Psychiatric Association (APA), and International Society for Traumatic Stress Studies (ISTSS), guidelines present medications and psychotherapy as equivalent first-line treatments (42). However, psychotherapies tend to be more heavily relied upon and produce longer therapeutic results compared to pharmacotherapy alone. The reason behind this is associated to the idea that pharmacotherapy largely masks the symptoms of PTSD rather than addressing the “conditioned fear responses” to previous traumatic stimuli (42). Finally, there are less-common treatments for PTSD including categories of alternative and complementary medicine as well as TMS.

#### Trauma-Based Psychotherapy

The most strongly supported forms of psychotherapy used in treatment of PTSD is trauma-focused therapy. Trauma-focused therapies for PTSD include, “a variety of techniques most commonly involving exposure and/or cognitive restructuring (e.g., Prolonged Exposure, Cognitive Processing Therapy and Eye movement Desensitization and Reprocessing)” (43). Traditionally, patients are exposed to past traumatic events and situations to identify their level of stress response. These events are then paired with learned skills and coping mechanisms that can alleviate symptoms of PTSD and alter their stress response.

Eye movement desensitization and reprocessing (EMDR) is a form of exposure therapy that combines a cognitive component as well as self-monitoring techniques. EMDR therapy involves an eight-phase treatment approach that includes the reprocessing phase where, “dual attention stimuli in the form of bilateral eye movements, taps, or tones” are used during exposure to traumatic events or memories to reassess how the patient deals with these scenarios (44). EMDR has been found useful in treatment of nightmare disorders associated with PTSD as this therapy improved symptoms and quality of sleep (45).

Another form of therapy commonly used is stress inoculation training (SIT) and is commonly used as a first-line alternative therapy to trauma-based therapy and is equally efficacious (43). SIT involves applying breathing techniques, relaxation techniques, and other forms of cognitive restructuring when faced with stressful or traumatic reoccurrences and memories.

#### Pharmacotherapy

Pharmacotherapy is used in the treatment of PTSD and while a number of off-label drugs are used in treatment, only two are approved by the FDA which are paroxetine and sertraline (46). The first line pharmacotherapy for PTSD includes selective serotonin reuptake inhibitors (SSRIs) and serotonin and norepinephrine reuptake inhibitors (SNRIs). More specifically, a meta-analysis study of 37 randomized placebo-controlled trials showed that paroxetine, sertraline, and venlafaxine were superior in symptom relief of PTSD compared to placebo (47). Furthermore, a *post-hoc* analysis suggests venlafaxine is effective for both men and women across all subtypes of trauma while paroxetine or sertraline were not effective across multiple subgroups (48). Buspirone is another medication, which works on the serotonin system, that could be used to treat PTSD. An open trial of 8 patients was performed which showed that 7 out of 8 of the patients had a significant reduction in symptoms as measured by the Structured Interview for PTSD and the Beck Depression Inventory (49).

The off-label drugs included in PTSD treatment range from tricyclic antidepressants like amitriptyline, imipramine to other like mirtazapine, nefazodone, phenelzine, and antipsychotics like risperidone and olanzapine (47, 50, 51). Prazosin is an alpha-1 adrenergic receptor antagonist that can be used in nightmare symptoms associated with PTSD (45). A placebo-controlled study showed that prazosin, “increased total sleep time, increased REM sleep time, and increased mean R period duration without alteration of sleep-onset latency” (52). Nefazodone is an antidepressant with a structure that is completely different from SSRIs, SNRIs, tricyclic antidepressants or monoamine oxidase inhibitors. It has been shown in one study to decrease depression, decrease intrusive symptoms, and improve sleep in patients with treatment refractory PTSD (53). In another study, nefazodone also was found to not only decrease depressive symptoms but it also improved global subjective sleep quality and a reduction in nightmares (54).

Ketamine is another off-label drug that is being studied for the treatment of PTSD. It is a drug that can be used for treatment resistant depression where it is noted to have a rapid onset with improvement of symptoms, however, the effect seems to wear off after 1–2 weeks (55). The rationale of using this medication in PTSD is the dampening of the NMDA receptors, however, it is thought that it could paradoxically increase anxiety with this mechanism of action as well (55). More studies will need to be performed to delineate both the drug's safety and efficacy as well as to develop treatment protocols for its use (55).

#### Alternative and Complementary Medicine

Complementary and alternative medicine (CAM) is used in treatment of PTSD for patients that either distrust modern psychiatric techniques or still have symptoms following traditional therapy (46). Complementary medicine includes practices like yoga and exercise which work together with traditional medicine to relieve symptoms. Alternative medicine techniques include acupuncture, animal-assisted activities or service animals, and alternative delivery methods of treatment such as virtual reality trauma-based therapy (46). While these methods are not commonly used, they have shown effectiveness in decreasing PTSD symptoms.

### Transcranial Magnetic Stimulation (TMS)

#### TMS Overview

TMS is a noninvasive brain-stimulating technique first introduced in 1985 (56). It has been used to help map the cortex with associated functions and as a therapeutic tool with increasing potential (56, 57). TMS uses the principles of Faraday's electromagnetism. An electric current is created that flows through a coiled circuit. This induces a magnetic field that is perpendicular to that current. The magnetic field travels until reaching another electrically conductive material to induce an electric current on that material (57).

In practice, one or two copper coils are connected to a capacitor that provides a changing electrical current through the coil. This coil is placed on the scalp at a particular location, depending on the treatment. The electric current produces a brief magnetic pulse. The magnetic field penetrates the scalp approximately 2 cm until reaching a conductive material, neurons in the cortex (57). An electrical signal is created to depolarize axons of the cortex. Axons are depolarized before the cell bodies of neurons due to the lower threshold of activation (58). This can lead to local changes in the cortex or secondary and deeper structures via neuronal subcortical pathways and neurotransmitter release (59, 60).

There are different modalities of TMS. The most commonly used is the repetitive magnetic transcranial stimulation (rTMS) where a regularly, repeated magnetic pulse is delivered to exert its effects (56, 61). rTMS can fire up to tens of pulses per second (62). Most recently, intermittent theta burst stimulation (iTBS) has been developed which delivers very high frequency stimulation over short periods of time. iTBS is generally considered a form of rTMS. Other used modalities are the simple-TMS, which uses a single magnetic pulse delivered over the cortex, and the coupled-TMS which uses two magnetic pulses that are separated by variable time intervals (63).

Currently, high frequency rTMS applied to the left DLPFC, is approved for use in treating major depressive disorder (MDD) in patients who have failed at least one medication trial (64). Patients showed improvement in mood, as well as improvements in working memory, episodic verbal memory, and language (56). Most recently, low frequency rTMS applied over the supplementary motor area has been approved for obsessive compulsive disorder (57).

Promising results have led to a rapid increase in research of TMS use in neuropsychiatric disorders. Therapeutic benefits have been shown in anxiety disorders, such as PTSD, generalized anxiety disorder, panic disorder, and social anxiety disorder. It can also be effective at reducing negative symptoms of schizophrenia and improving symptoms in neurodevelopmental disorders such as Tourette's and autism spectrum disorder, substance abuse disorders, brain damage, neurodegenerative disorders such as Alzheimer's and Parkinson's disease, motor stroke, and fibromyalgia (64).

TMS therapy has advantages over other forms of therapy due to its relative safety and fewer adverse effects. However, clinicians should be aware of adverse effects and contraindications. The most common side effects are headaches, neck pain, and local pain at the stimulation site (65). Pain is thought to be related to stimulation of superficial nerves and due to uncomfortable positioning during treatment. Headaches may be caused by increased blood flow from local scalp stimulation (65). Serious adverse effects are rare with seizures being the most commonly reported from 0.1–0.6% (57, 61). The risk of seizure is greater with high-frequency treatments and those with intense protocols. Other neurological conditions and medications can lower the threshold for seizures (66). Therefore, TMS is contraindicated in patients with a history of epilepsy and medications that lower the seizure threshold should be titrated down or stopped. Other absolute contraindications of TMS include any metallic devices or implants that are in close contact to the coil. Conducting substances can induce eddy currents when near the magnetic coils (67). Therefore, cochlear implants are contraindications for TMS. There is no evidence of cognitive impairment in TMS, unlike in electroconvulsive therapy (68). TMS is a newer therapy with more research needed for sufficient data of possible long-term adverse effects.

TMS is a not a one-size-fits all and there are a number of variables to account for when applying the technique. There are inter-individual differences in anatomy and pathology which makes application of this therapy difficult (56). Several studies have shown variability in healthy subjects in responses to all TMS protocols (58). More research must be completed to demonstrate mechanism of action and reasons for variable response before TMS can be used as an approved therapeutic tool in other neurologic diseases (66).

## Neurobiology of TMS

TMS has various applications and can produce motor responses, alteration in memory, and modify executive function. Ongoing investigations are looking into how such changes can be explained physiologically. It has been observed that certain modalities and settings are associated with reproducible findings specifically with memory formation and corticospinal excitability (69). Predictable modifications are observed as well as other downstream effects due to the presence of neuronal network connections. Knowledge of particular neural networks should be used strategically. Currently, TMS is utilized primarily focusing on superficial, cortical regions. Since deeper brain structures are also affected, they have the potential to be used as a target for modulation once neural networks are better understood (70).

### The Motor Response and Establishing Target Regions

TMS can be used to create muscle contraction. Stimulation of the motor cortex using TMS pulses provides a simple example of how a magnetic field located transcranially can produce an observed response. In the original study, trains of TMS pulses were applied over the motor cortex and the hand was observed for muscle twitches of the targeted muscle, the first dorsal interossei. This stimulation preferentially activated interneurons oriented in a plane parallel to the brain surface, this created downstream effects by transsynaptic activation of pyramidal cells which descended and projected onto spinal motor neurons (the corticospinal tract). Activation of the motor neurons would lead to contraction of the target muscle and was either observed as muscle twitches or measured with electromyography of the muscle belly (69). This observed motor response is used to help locate the DLPFC which usually lies around a 5-6 centimeters in front of the primary motor cortex. However, this targeting method frequently was not able to localize the DLPFC as there may be significant variability in functional localization for this area within the PFC as a whole (70). Currently, localization works best when MRI is used to directly target and visualize the region of interest (71).

### High vs. Low Frequency Stimulation

Depending on the settings and type of stimulation, TMS can affect neuronal activity in a variety of ways. Two of the most studied variables include high vs. low frequency, and intermittent vs. continuous burst firing. Low frequency stimulation is <1 Hz and has been found to decrease cortical excitability, while high frequency stimulation is >1 Hz and increases cortical excitability. This is created by a magnetic field that passes through the scalp and skull creating changes to cortical and subcortical activity in specific brain networks (2). Additionally, along with altering TMS frequency, the duration of application can be altered which can directly alter synaptic strength and memory formation. For example, long-term depression (LTD) can be induced when low frequency stimulation is used for long periods, while high frequency stimulation for short periods will induce long-term potentiation (LTP). LTD is defined as weakening of the synapse, while LTP is strengthening of the synapse. These changes are mediated with TMS due to stimulation of the pathways and circuit changes that induce plasticity (69). The current understanding behind this mechanism is explained by the relationship between LTP and NMDA receptors. NMDA receptors are cation channels that lead to activation of a calcium sensitive signaling pathway. This activation leads to downstream effects at the level of pre- and post-synaptic neurons and strengthens synapses (72). Contrarily, LTD also works through the NMDA receptor, although induced through a different calcium flux through the channel. LTP is mediated via fast and large increases in calcium concentration, while LTD is facilitated through a slow and small increase in calcium concentration following long periods of low frequency stimulation and creates different changes to the synapse (69). These findings exemplify how TMS settings can be altered to alter memory and plasticity, which has clinical utility in PTSD where individuals are significantly impaired by miscoded, intrusive memories.

### TMS Targets for Mood Disorders

The DLPFC is an important target in TMS as it is a core component of a number of neural networks involved with cognitive and behavioral functions. Using both high and low-frequency rTMS, the right DLPFC has been found to be a promising therapeutic target in mood disorders. Both high and low frequency were found to provide benefit to patients with PTSD even though they have opposing mechanisms of action, as high-frequency rTMS induces cortical excitability and low-frequency rTMS is inhibitory (73). This contradictory finding is explained by the different effects produced by high-frequency rTMS at the cortex and downstream changes to the amygdala. This theory relies on the current understanding that PTSD involves a hypoactive PFC and hyperactive amygdala. When high-frequency rTMS is applied to the right DLPFC in PTSD patients, excitatory effects are produced at the underlying hypoactive cortical tissue, this activation leads to subsequent indirect inhibition of the amygdala. Modifying the activity level of the DLPFC and amygdala assists in the treatment of PTSD by altering memory consolidation and modulating fear-related emotional responses. It is not fully understood how low frequency rTMS produces favorable changes to PTSD symptoms, however, one model explained that therapeutic effects are observed due to partial inhibition of lateralized right-sided hyperactivity of the DLPFC (73).

## Clinical Studies: Efficacy and Safety

### Efficacy

Overall, TMS therapy has been suggested as an effective treatment in PTSD patients. Results of two meta-analysis showed that TMS therapy provides an overall therapeutic effect in PTSD patients (74). In one study, levels of active distress decreased along with insomnia (a major symptom of PTSD) although intrusive memories, avoidance and hypervigilance did not (18). Another study showed that using repetitive rTMS compared to sham rTMS treatment produced significant reductions in PTSD symptoms from baseline (13).

Repetitive TMS isn't the only type of stimulation that has shown beneficial effects. In fact, a study conducted by Philip et al. used individual theta-burst TMS (iTBS) and compared this to sham treatment of iTBS in fifty veterans with PTSD (11). Although these researchers used a different sub-type of TMS therapy, results showed that at 2 weeks patients had significantly improved social/occupational function and improved symptoms compared to sham treatment (11). It is worthwhile to note that two of the studies listed above are limited in following the duration of effects of treatment. At 2 weeks of treatment, the study by Philip et al. showed iTBS reduced symptoms from baseline but longer effects of treatment was not followed. The same limitation occurred with the study conducted by Ahmadizad et al. Conversely, this limitation has been investigated by Watts et al. where this group of researchers looked at rTMS delivered to right DLPFC compared to sham treatment and followed the effects up to 2 months post-treatment (13, 14). The results of the study suggest that although rTMS was beneficial in reducing PTSD symptoms initially, effectiveness degraded throughout the 2 month period of follow up once treatment had ceased (14). A contradictory conclusion is made by Boggio et al. who showed that effects from rTMS lasted and were significant up to 3 months following treatment (15). This raises questions whether TMS therapy needs to be applied periodically or until symptoms have ceased and if effectiveness is scaled in post-treatment months.

Another variable that has been addressed is the location of which TMS therapy is applied. The double-blinded, placebo-controlled phase II trial study by Boggio et al. looked at 30 PTSD patients and examined the effects of TMS treatment to both the left and right DLPFC and found that right-sided rTMS induced a larger effect compared to left-sided (15). Additionally, these researchers found that improvement in both avoidance and hyperarousal was larger for right-sided rTMS treatment compared to left while reexperiencing symptoms were similar in either side of treatment. Furthermore, although application to the right DLPFC has typically been found to be more efficacious compared to left, the use of bilateral application (both right and left DLPFC cannot be ruled out (13). Left sided application may be beneficial in certain instances as Rosenberg et al. attempted to study the left DLPFC because previous reports suggested that this area is more associated with improvement of mood symptoms (18). Regardless of laterality, TMS application to the DLPFC seems to produce the most effective results in treatment of PTSD.

Effectiveness of TMS therapy is also partially determined on the frequency of electromagnetic stimulation that is applied during each session, measured in hertz (Hz). It has been found that even low-frequency stimulation at 1 Hz has proved beneficial in patients during acute treatment (17). Moreover, Kozel et al. failed to show that there was a statistical difference in improvement in PTSD symptoms in the 1 Hz treatment group compared to the 10 Hz treatment group. This is contradicted by a double-blinded controlled study by Cohen et al. in 2004 that suggested that high-frequency (10 Hz) rTMS group showed greater therapeutic effects than the low-frequency (1 Hz) rTMS group (16). Both groups of researchers concluded that further work with a larger sample population would be beneficial to determine more accurate frequencies of stimulation but found that rTMS therapy is effective regardless of frequency level.

Other studies applying TMS have looked at combination treatment with certain types of therapy such as prolonged exposure (PE) therapy and cognitive process therapy (CPT). A prospective, randomized, double-blinded, active sham-controlled design study looked at the effects of rTMS sessions prior to PE therapy (12). The findings suggested that patients had no difficulty tolerating both rTMS and PE therapy but were inconclusive of whether combination therapy was beneficial. Additional studies with larger sample sizes would be needed to assess further. When rTMS combined with CPT was applied, there was significantly greater PTSD symptom reductions early in treatment and 6 months post treatment (17). Both of these studies reinforce the positive effect of TMS in PTSD patients but fail to conclude that combination therapy is beneficial for this population.

### Safety

While TMS therapy appears to be effective in treating patients with PTSD symptoms, safety remains a concern. In more recent studies mentioned above, a set of guidelines suggested by Wasserman were used (75). Wasserman suggested following specific criteria regarding frequency, intensity, duration, and repetitive pulse parameters based on motor evoked potentials (MEP) to maintain safety and efficacy. This data was based on side effects associated with rTMS that included seizures, headaches, scalp pain at location site, effects on hearing, and effects on mood.

### Neurological and Neuropsychological Effects

Seizures are a known side effect that have occurred in patients using TMS therapy. In one study, one healthy volunteer had a secondary generalized seizure occurred causing neurological changes that resolved within the postictal state while all other participants (n = 10) remained unchanged (76). Before Wasserman released his protocol for safety of rTMS, seven seizures had been reported. The majority of patients had no recurrence and did not have any lasting sequelae (75). Furthermore, cognitive performance improved in all patients following rTMS as well as reaction time and memory (76). This is supported by Boggio et al. who found that TMS therapy to both the right and left DLPFC is safe and not associated with declines in cognition (15).

### Headaches

Headaches are a common side effect associated with rTMS therapy as stimulation occurs and affects the nerves and muscles of the skull at the application site. This causes a discomfort that can be significant for individuals and lead to persistent muscle tension-type headaches (75).

Frequency and intensity are the most common factors associated with headaches from therapy (77). This is substantiated in recent studies as the most common side effect (11). Treatment is simple with mild analgesics and will subside usually without persisting longer term effects (75).

### Scalp Pain

Eddy currents induced in metal surface EEG electrodes located near a stimulating coil can cause heating and skin burns during rTMS which can be uncomfortable for most patients (78). Scalp pain at the location site is noted as one of the most common side effects of TMS therapy (11). Following the use of Wasserman's safety protocols, this rarely occurs (17).

### Effects on Hearing

Counter et al. noted that exposure to single-pulse TMS lead to permanent increases of auditory threshold and transient increases in human subjects (79). This was contradicted in another study where no hearing loss was noted in subjects exposed to single-pulse TMS (80). Regardless, foam earplugs have been suggested as a remedy when using TMS therapy and have limited hearing issues as a side effect.

### Effects on Mood

Speech arrest and laughter has been observed in healthy subjects receiving rTMS stimulation (77). Other studies have noted a similar coexisting relationship between these two side effects in epileptic and hemiparetic patients receiving TMS as well (67). Crying has also been observed in TMS therapy although this is rare (75). Overall, effects on mood are transient and fail to persist long term.

Wilke's et al. looked at the impacts of rTMS on refractory depression and Comorbid PTSD symptoms in veterans (19). This was a retrospective chart review of 77 patients who received rTMS between January 1, 2010 and October 31, 2016. The patients received rTMS for 6 weeks along with weekly psychiatric assessments which included completed Beck's Depression Inventory (BDI) and PTSD checklist (PCL). 52% of patients had copleted trials of three or more antidepressants prior to treatment. Both BDI and PCL scores were significantly lower at the end of rTMS treatment when compared to the baseline scores obtained pretreatment (19). The mean differences for BDI and PCL were significant at 15, 30, and 45 days after rTMS treatment was initiated (*p* < 0.001). 44% of patients had a reduction greater than or equal to 10 points on the BDI and 38% experienced a reduction of the same amount on PCL (19).

### Meta-Analysis and Systematic Reviews

A meta-analysis performed looked at PTSD mood outcomes with the use of TMS (81). This was a literature search of major online databases from inception to September of 2020 which primarily searched for studies using TMS to treat PTSD. The authors found an overall effect size of d = 1.17, 95% CI [0.89–1.45] in regards to TMS as a treatment for PTSD (81). An analysis of moderators shows that was a significantly larger treatment effect for high-frequency TMS (d = 1.44) compared with low frequency (d = 0.72) with a *p*-value of 0.006. There was no significant difference between whether TMS targeted the DLPFC and right DLPEC. Larder treatment doses were not associated with stronger treatment effects (81).

A systematic review of studies was performed by Belsher et al. in 2021 which looked at the use of rTMS for PTSD (20). This review looked at 13 studies with 549 participants and the authors compared the effects of rTMS verus sham, high-frequency verses low-frequency rTMS on posttreatment PTSD scores using calculated standardized mean differences (SMD). The authors concluded that at posttreatment rTMS was superior to shame in reducing PTSD symptoms (SMD = −1.13 95% CI [−2.10–−0.15]) (20). However, they also concluded that the quality of their analysis was rated as very low due to small sample sizes, treatment heterogeneity, inconsistent results, and an imprecise pooled effect. High-frequency rTMS was associated with slightly improved outcomes compared to low-frequency rTMS on PTSD, however, this was also imprecise. They concluded that further research is required to further delineate the effects of rTMS on PTSD (20). Table 1 is a summary of the studies discussed in this section.

**Table 1:** Clinical Safety and Efficacy.

**Author (Year)**	**Groups studied and intervention**	**Results and findings**	**Conclusions**
Philip et al. (11)	Fifty veterans with PTSD received 10 days of sham-controlled iTBS (1,800 pulses/day), followed by 10 unblinded sessions. Studiers measured retention rates, changes in PTSD symptoms (clinical and self-rated), quality of life, social/occupational function, and depression at 2 weeks of treatment.	At 2 weeks, iTBS was significantly associated w/ improved social/occupational function along w/ improved depression compared to sham treatment. Moderate nonsignificant effect sizes were observed on self-reported PTSD symptoms. One-month outcomes indicated superiority of active iTBS on clinician and self-rated PTSD symptoms, depression, and social/occupational function.	iTBS appears to be a promising new treatment for PTSD with clinical improvements occurring early in treatment. This suggests duration and time course of iTBS therapy as further studies.
Fryml et al. (12)	A prospective, randomized, double-blinded, active sham controlled design combined weekly sessions of rTMS and standard PE for 5 weeks. 8 military veteran patients received full course of protocol-driven PE therapy and then placed in rTMS or sham group. The goal was determined improvement of PTSD following 5- week course.	Of 12 consented patients, 8 completed therapy with a dropout rate of 34%, suggesting patients had no difficulty tolerating addition of rTMS to PE therapy. Clinician-Administered PTSD Symptom scores reflected a general nonsignificant trend toward improvement, while comorbid patients with MDD experienced significant antidepressant benefit with treatment.	The pilot study demonstrates the safety and feasibility of rTMS delivery to PTSD patients and assesses the need for studies with larger sample sizes to assess treatment outcomes.
Ahmadizadeh et al. (13)	Randomized controlled trial of bilateral, unilateral right, or sham rTMS treatment on Sixty-five patients w/ combat-related PTSD symptoms.	Patients demonstrated significant PTSD symptom reduction in the bilateral group compared to the sham group but no significant difference between bilateral and unilateral right groups. The unilateral right group compared to the sham group showed greater symptom reductions from baseline.	Bilateral and unilateral right rTMS therapies are superior to sham rTMS but does not support the hypothesis that bilateral rTMS is more effective than unilateral right sided rTMS.
Watts et al. (14)	Twenty PTSD patients subjected to 10 rTMS sessions delivered at 1 Hz to DLPFC or 10 sham rTMS sessions to same area. A blinded rater assessed PTSD symptoms before treatment, after treatment, and during a 2 month follow-up period.	TMS delivered at 1 Hz to right DLPRC results in statistically and clinically significant improvements in core PTSD symptoms compared with sham treatments. Effectiveness degraded during the 2 months follow up after treatment had stopped.	This blinded sham controlled trial supports the efficacy of 10 sessions of right DLPRC rTMS delivered at 1 Hz for the treatment of PTSD symptoms.
Boggio et al. (15)	A double-blind, placebo-controlled phase II trial with 30 PTSD patients randomly assigned to receive active 20 Hz rTMS of right DLPFC, active 20 Hz rTMS of left DLPFC, or sham rTMS over 10 daily session spanning 2 weeks. A blind rater assessed severity of core PTSD symptoms before, during, and after treatment protocol.	Both the 20 Hz rTMS of left and right DLPFC induced a significant decrease in PTSD symptoms, however right rTMS induced a larger effect compared to the left rTMS. Additionally, there was improvement in mood with left rTMS and a reduction in anxiety following right rTMS. These effects were long lasting and still significant at the 3-month follow-up.	Modulation of prefrontal cortex can alleviate the core symptoms of PTSD and suggests that high-frequency rTMS of right DLPFC might be the optimal treatment strategy.
Cohen et al. (16)	A double-blinded controlled study with 24 PTSD patients received either rTMS at 1 Hz, rTMS at 10 Hz, or sham rTMS administered over 10 daily sessions for 2 weeks. PTSD symptoms were assessed before, during and after treatment protocol.	The 10 daily treatments of 10 Hz rTMS over the right DLPFC had therapeutic effects for PTSD patients and improved core symptoms. Additionally, high-frequency (10 Hz) rTMS over the right DLPFC alleviated anxiety symptoms in PTSD patients.	This study suggests that over 10 daily sessions of rTMS (10 Hz) over the right DLPFC for 2 weeks has greater therapeutic effects than slow-frequency (1 Hz) rTMS and sham stimulation.
	Combat-related PTSD patients were randomized using a 1:1 ratio in parallel design to active (rTMS+CPT) vs. sham (sham+CPT) rTMS just prior to weekly CPT for 12-15 sessions. rTMS was positioned over the right DLPFC (1 Hz). Blinded raters evaluated patients at baseline, after the 5^th^ and 9^th^ treatments, and at 1,3 and 6 months post-treatment.	103 participants were randomized to either active rTMS or sham rTMS. 60% completed treatment and 59% completed 6-month assessment. The rTMS+CPT groups showed greater symptom reductions from baseline across CPT session and follow-up assessments.	The addition of rTMS to CPT treatment compared to sham treatment with CPT produced significantly greater PTSD symptom reduction early in treatment and was sustained up to 6 months post-treatment.
Kozel et al. (17)	Combat-related PTSD patients were randomized to right prefrontal rTMS (1 Hz) vs. rTMS (10 Hz). Treatments occurred 5 days a week for 6 weeks with 3-week taper. Follow-up evaluations were performed at one month and three months.	Both groups (1 Hz and 10 Hz) had significant improvements in PTSD and depression scores from baseline to the end of acute treatment. The 10 Hz group demonstrated significant improvement in function while the 1 Hz group did not. A significant advantage for either the 1 Hz or 10 Hz frequency group on any of the scales was not be demonstrated.	Treatment using rTMS to the right DLPFC in combat-related PTSD patients significantly improves symptoms. Further work to determine whether low or high frequency rTMS is superior in larger populations would need to be demonstrated.
Rosenberg et al. (18)	Twelve patients with comorbid PTSD and MDD underwent rTMS to left frontal-cortex as an adjunct to antidepressant medications.	75% of patients had a clinically significant antidepressant response after rTMS. Improvements were seen in anxiety, hostility and insomnia but minimal improvements in PTSD symptoms.	This study shows that lDLPFC rTMS could be effective in treating depression associated with PTSD patients.
Wilkes et al. (19)	Retrospective chart review of 77 patients who received and completed rTMS treatment with refractory depression and PTSD symptoms rTMS was given for 6 weeks along with weekly psychiatric assessments which included the completion of BDI and PCL	52% at completed trials o three or more antidepressants 44% experienced a reduction of ≥10 points on BDI 38% experienced a reduction of greater than or equal to 10 points on PCL	rTMS may produce a reduction in symptoms of both depression and PTSD in patients with refractory depression and comorbid PTSD and may be a useful alternative to treatment
	Meta-analysis of studies regarding TMS for the treatment of PTSD Search included studies from major online databases from inception to September 15, 2020	Overll effect size of d = 1.17, 95% CI [0.89–1.45] for TMS as a treatment fot PTSd Significantly larger treatment effect for high frequency TMS (d = 1.44) compared with low frequency (d = 0/0.27) *p* = 0.006 No significant difereten was found between TMS targeting the left DLPFC and right DLPFC	TMS can be an effective treatment for PTSD but more research is required to understand the neurological mechanism of TMS on specific PTSD symptoms
Belsher et al. (20)	Systematic review of 13 studies including 549 participants Compared rTMS vs. sham, high frequency vs. low frequency rTMS on posttreatment PTSD scores	rTMS was superior when compared to sham in reducting PTSD symptoms (SMD = −0.13, 95% CI [−2.10−0.15]) High frequency rTMS was associated with slightly inporved outcomes when compar to low-frequency rTMS (SMD = −0.019, 95% CI {−0.19–1.00])	rTMS could be an effective treatment for PTSD quality of evidence was rated as very low due to small sample sizes, treatment heterogeneity, inconsistent results, and an imprecise pooled effect More research is required tfor this treatment

## Conclusion

Early studies have shown encouraging results for the use of TMS as a therapeutic tool in treating PTSD. Its use appears to be relatively safe with few adverse effects and is approved by the FDA for the treatment of refractory MDD. However, more data needs to be gathered to confidently use TMS as an approved treatment protocol for PTSD.

A limitation to applying this technique is that PTSD is not fully understood, as seen by the evolving definition of PTSD in the latest updates of the DSM. In addition, there are multiple theories that explain the underlying mechanism behind PTSD. These include dysregulation of the HPA axis, neural networks, and the relationship between DLPFC and amygdala in memory and fear learning. Although further research and understanding is needed, these theories are promising in the application of TMS as a treatment of PTSD.

To maximize therapeutic potential and minimize possible adverse effects, TMS parameters and delivery of the therapy for PTSD will need to be established. Deciding on the optimal target region and application settings will be required, as TMS has opposing effects when administered at different frequencies and pulse patterns. Additionally, as TMS will be used clinically, determining the number of doses and time of delivery will be required for creating an effective treatment regimen. Finally, more research is required in the investigation of PTSD and TMS to understand how the technology can be applied for treating the invasive symptoms of the disorder.

Along with understanding PTSD, the mechanism behind TMS also requires further investigation. Resistant depression has been treated with TMS and therefore it is worth applying the technology for the treatment of PTSD. Initial research has produced short-term successes, showing a reduction in symptoms and overall therapeutic effects in PTSD patients. However, many of the studies have been weak due to sample size or length of the study, with some repeat studies producing contradictory results. There is also no evidence out there regarding the concurrent use of psychotropic medications, which is considered part of the standard of care for PTSD, and with the use of TMS. More research will need to be done with larger sample sizes over longer periods of time to strengthen results.

## Author Contributions

All authors listed have made a substantial, direct, and intellectual contribution to the work and approved it for publication.

## Conflict of Interest

OV was employed by Valley Anesthesiology and Pain Consultants. The remaining authors declare that the research was conducted in the absence of any commercial or financial relationships that could be construed as a potential conflict of interest.

## Publisher's Note

All claims expressed in this article are solely those of the authors and do not necessarily represent those of their affiliated organizations, or those of the publisher, the editors and the reviewers. Any product that may be evaluated in this article, or claim that may be made by its manufacturer, is not guaranteed or endorsed by the publisher.
